# Relationships of Adolescent and Young Couples with Violent Behaviors: Conflict Resolution Strategies

**DOI:** 10.3390/ijerph18063201

**Published:** 2021-03-19

**Authors:** Noelia Aguilera-Jiménez, Luis Rodríguez-Franco, Paloma Rohlfs-Domínguez, Jose Ramón Alameda-Bailén, Susana G. Paíno-Quesada

**Affiliations:** 1 Department of Social, Developmental and Educational Psychology, University of Huelva, 21071 Huelva, Spain; noelia.aguilera@dpee.uhu.es; 2 Department of Personality, Assessment and Psychological Treatment, University of Seville, 41018 Seville, Spain; lurodri@us.es; 3 Department of Psychology and Anthropology, University of Extremadura, 10003 Caceres, Spain; palomaroh@unex.es; 4 Department of Developmental and Educational Psychology, University of Basque, 48940 Basque, Spain; 5 Department of Clinical and Experimental Psychology, University of Huelva, 21071 Huelva, Spain; alameda@uhu.es

**Keywords:** level of agreement or concordance, dating violence, adolescents, young people, conflict resolution strategies, violent behaviors

## Abstract

Violence in adolescent and young couples is a major issue given its high prevalence and the serious consequences that it brings. For this reason, this research has stated two main objectives. In the first place, to ascertain the level of agreement between both members of the couple both with regard to occurrence and frequency of violence. Second, to ascertain the level of agreement on the frequency of use of conflict resolution strategies in problematic situations in 141 heterosexual couples. The age of the sample was between 17 and 30. The tools used were the DVQ-R questionnaire and the Spanish adaptation by Bonache, Ramírez-Santana, and González-Mendez (2016) of the Inventory of Conflict Resolution Styles (CSRI)The results indicate that of the 141 couples in the sample, 112 were identified as violent, thus indicating a high prevalence of violence within their partner relationships. Regarding the levels of agreement and accordance, statistically significant discrepancies are reflected in the perception of violence between men and women, analyzing both roles (aggression and victimization). Finally, also noteworthy is use of the strategy of negative involvement in conflicts, with significant differences in relation to sex; it is the girls who make the most use of this strategy, and the high level of agreement on the frequency of problem-solving is reflected on that strategy.

## 1. Introduction

According to the World Health Organization (2013) [1] (p. 1) intimate partner violence is understood as: “any behavior in an intimate relationship that causes physical, psychological, or sexual harm. It includes acts of physical aggression (slaps, blows or kicks), psychological abuse (intimidation, disdain or continuous humiliation), forced sexual interactions, and controlling behaviors (isolation from family and friends, controlling of movement and restrictions on access to information or help).”

Partner violence is a problem of clinical and social interest that has been present in our society as clearly shown in the scientific literature for decades [2]. All of which has led to a considerable increase in studies on the adolescent and young population given the importance of the topic, the impact on health, and the interest in prevention during this stage when dating and relationships begin [3,4]. These studies aim to identify the presence of the different types of violent behaviors, as well as their seriousness at a young age and, above all, to discover the associated risk factors [5]. 

### 1.1. Violence in Adolescent and Young Couples’ Relationships

Specifically, violence in dating relationships can be present in anyone independently of their sexual orientation. In fact, one of the clear conclusions that has been reached is that violence is a human and not just a gender phenomenon, as there are many ways of exerting it [6,7]. Currently, the research that studies interpersonal relationships of the younger couples describes scenarios that are different from those of a few years ago and from those of adult couples [8,9]. With regard to heterosexual couples, these are characterized by high indices of prevalence of violence both in men and women. Moreover, the literature also highlights that they are higher in young couples than in adult couples [10,11,12,13] giving rise to serious consequences with physical and psychological sequelae [14,15].

To date, one vision of violence has mainly been studied which has helped us to understand this problem, but which presents certain limitations, as there are others with different characteristics which are very frequent in dating relationships like for example, its two-way nature or mutual behaviors, in which both partners can acquire the two roles present in a relationship: both the man and the woman can be partner abusers and, consequently, the victim of abusive behavior in the same relationship and in the same scenario. Moreover, several authors have begun to focus on the study of the two members of the couple, analyzing the actor and the couple to thus discover the scope of their victimization and aggression [8,12,16,17,18,19,20,21,22]. These studies have found low levels of agreement between the two members of the couple regarding their perceptions on occurrence and frequency of different types of violence. In contrast to these studies, the present study additionally examines occurrence of use of maladaptive problem-solving strategies as well as the level of agreement between the partners of the couples regarding their perceptions on the frequency of use of those strategies. This variable is important because it has an impact on whether the couple is able to stop occurrence of violence, that is, the more maladaptive problem-solving strategies are used, the higher the occurrence of violence. To the best of our knowledge, it is the first time that that variable is included into a research study of the field. 

### 1.2. Prevalence of Violent Behaviors in the Relationships of Young and Adolescent Couples

In relation to the most dominant factors of violence, the research highlights the frequency of psychological aggression [4,20,23]. Specifically, Muñoz-Rivas, Graña, O’Leary, and González (2007) [11] found percentages of up to 80% in psychological violence. García-Carpintero et al., 2018 report figures of 12% in sexual aggression and 14.1% in physical violence. Bearing in mind the possible differences by sex, authors like de la Villa, García, Cuetos and Sirvent (2017) [24], indicate that women are the ones who initiate the abusive behavior, and even use it to a greater extent. On this basis, women make more use of psychological violence, but when it is a case of more severe violence like physical violence, then it is the men who present the highest indices [25,26]. Furthermore, the findings of Herrero et al. (2020) [8], the results of the analysis of both partners on mutual violence, also underline that psychological violence is the most common, and physical and sexual aggression show a lower prevalence. According to them, the couples are most aggressive when there is a dynamic of mutual violence in the relationship.

### 1.3. Agreement on Violence within the Relationships of Adolescent and Young Couples

Another key point in the investigation of adolescent and young couples consists of the concordance that exists between both partners of a couple regarding the agreement of the acts of victimization and perpetration of violence. In this regard, little scientific research has been done, and the results remain controversial. Moreover, most of the studies have included only adult participants. The present study aims to bridge this research gap. Regarding the studies with adults, results indicate a low agreement between the two partners of the couple when it comes to exercising physical violence toward the other [27,28]. This can be explained because men indicate that they are victims of physical violence in a higher extent that their female partners recognize they are, and, likewise, women report that they are victims of physical violence in a higher extent than their male partners say they are [29]. Regarding the studies with young participants, results show high levels of physical violence, but a low agreement on the occurrence of such violence and inclusion of sexual behaviors [30]. On the other hand, other studies with adolescent and young population, show, with low-to-moderate reliability levels, a high level of disagreement between both partners of the couple on psychological violence [31,32]. This is, according to these studies, because women report more violence than men [31,32]. However, two additional studies [33,34] showed a high level agreement between both members of the couple on psychological violence. According to these studies, adolescents report psychological abuse because they do not perceive it as violence and thus it is acceptable to them [33,34]. The low agreement between the partners of the couple on physical and psychological violence has been attributed, however, to social desirability [31,34].

### 1.4. Conflict Resolution Strategies in Adolescent and Young Couples

Continuing with the progress made in the study of couple violence, many authors investigate violence in relationships oriented toward the tackling or management of situations of conflict in the younger population; mainly to understand the scope of the strategies and resources managed by the adolescents and young people in situations of couple conflicts [35]. We understand that conflicts can be motives for breakups and distress, but they are also habitual elements in the relationship [14,36]. Obviously, every individual presents different coping strategies to resolve or reduce problems [37]; however, there are studies with the young population that indicate a strong correlation between the young people who show a behavior of emotional dependence toward their partners and more aggressive coping, with a negative correlation when the variable is the positive strategy facing conflicts. Specifically, violence in younger couples is related to emotional dependence and, finally the latter with more aggressive strategies toward the partner [38,39]. These studies show that the young people have difficulties in coping with conflicts because of inadequate emotional regulation or incapacity to perceive the consequence of their acts. For them, violence is habitual in situations of conflict, which combined with inexperience and lack of abilities in interactions, means that they understand violence as the normalized pattern within the relationship and this becomes a risk factor for future relationships as they understand intimate relationships as including conflicting and violent dynamics [16,31,40,41,42,43,44], which, as mentioned, affect their wellbeing and health.

### 1.5. Interest in the Study of Relations in Adolescent and Young Couples

Bearing in mind the prevalence of violence in both sexes when it presents in a two-way or mutual manner, aggressive behaviors of women toward men within couple relations is an under-recognized or invisible phenomenon, which is in no way socially alarming and, even less, a social problem [45], in spite of the fact that community data are indicating that it is a phenomenon that is increasing and continues to be ignored [46,47]. The fact of disregarding that within interpersonal relationships it is not only a case of violence on the part of the man toward the woman, limits us and prevents us from obtaining a complete and differential description of the violence generated in intimate relationships, whether heterosexual, homosexual, or bisexual. If we really stop dodging the multiple forms in which violence is present, public and social policies will be able to establish a better response. To summarize, the numerous studies that we have worldwide on the use of violence in adolescent and young couples, show us the frequency with which this phenomenon occurs. Furthermore, it should again be underlined that violence in dating shows different forms and directions, that is, there is not only a man or woman aggressor and a man or woman victim. It is also necessary to tackle the normalization of dysfunctional relation dynamics, as this has consequences above all in the identification, recognition, and awareness of being immersed in an abusive relationship. This, in consequence, is an obstacle that must be overcome, and the best form of prevention is to impede any indicator at the beginning, as those couples who have dysfunctional relationship dynamics have recourse to violence as a means of communication and conflict management entering into an escalation of violence, and thus, causing adverse effects on their health [48,49].

Ultimately, dating violence within adolescent and young couples is an issue of particular interest for several reasons. First, it is well-known that there is a high prevalence of abusive situations within adolescent and young couples that have not yet begun to live together, psychological aggressions being the most frequent form of abuse. Second, we cannot forget the extremely serious physical and psychological consequences that partner violence has on the members of a couple. Third, it is well-known that the partner violence that is inflicted and suffered during adolescence and youth can continue during adulthood. Finally, adolescents and young people often do not perceive the partner violence that they have inflicted and suffered. The consequence is that they do not seek help, and thus partner violence commonly persists over time. For these reasons, the study of the agreement between members of adolescent and young couples on their perceptions of inflicted and suffered partner violence is warranted.

On the other hand, the study of perceived partner violence has been traditionally carried out by taking into account only the perception of only one of the members of the couple with the consequent biases in the results obtained. Our study, however, includes the perception of both members of the couple (actor-partner) [8,31] in order to achieve a greater reliability in the data obtained regarding perceived prevalence of dating violence.

Given this background, this research has the following objectives. First, to ascertain young people´s perceived partner violence in young population according to sex and different types of violence. Second, to ascertain the level of agreement between the members of adolescent and young couples in both the perceived occurrence and frequency of partner violence. Third, to ascertain the level of agreement between members of adolescent and young couples on the perceived frequency of use of conflict resolution strategies when it comes to facing problematic situations by both partners. This more in-depth knowledge of dating relationships with violent behaviors will help us in our daily work, as when we have the two points of view, we can develop complete and suitable interventions. In addition, results of the present study may help contribute promoting healthy and satisfactory relationships that are based on the absence of violence.

On the basis of the described objectives, the following specific exploratory objectives were established: 1. High rates of dating violence: Both members of the couple use violence and show both the role of aggressor and the role of the victim. 2. Low levels of agreement between the two members of the couple regarding their perceptions on occurrence and frequency of violence. 3. High agreement between the members of the couples regarding their perceptions on the frequency of use of maladaptive problem-solving strategies in the role of perpetration.

According to those objectives, we expect to find: 1. High rates of violence in young couples, and both members of the couple use violence regardless of sex, and, consequently, both members take both the role of aggressor and the role of victim. 2. Low levels of agreement for the occurrence of violence; a higher level of agreement for the frequency of physical and/or sexual nature, and a lower level of agreement for violence of psychological nature. 3. Use of maladaptive conflict-resolution strategies when exerting violence.

The present study is a continuation of previous research i.e., [8,12,16,17,18,19,20,21,22], but includes two new variables: perception of the two members of the couple and used problem-solving strategies.

## 2. Materials and Methods

### 2.1. Participants

The sample comprised 282 subjects, 141 heterosexual couples who were in a relationship at the moment of the assessment from the provinces of Huelva and Extremadura (Spain). Of the total sample 141 (50%) were women and 141 (50%) were men. The age of the participants was between 17 and 30 years, the women’s mean age was 22.34 and the men’s 22.32. With regard to the educational level, all had completed compulsory secondary education, high school, vocational training or university studies. Specifically, in the women’s sample 77 (54.6%) had completed compulsory secondary education or vocational training, and 64 (45.4%) university studies, while of the men 95 (67.3%) had completed compulsory secondary education or vocational training and 45 (31.9%) university studies. Thirty-one point nine per cent of the women indicated that they had a job compared to 68.1% who did not at the time of assessment. In the case of the men 47.5% reported having a job and 52.5% did not. Lastly, regarding the religious beliefs of this sample of couples, 37.6% of the women and 44.7% of the men considered that they were not at all religious. The sociodemographic profile is described in more detail in Table 1.

### 2.2. Measurement Instruments

**Sociodemographic characteristics.** Information was collected related to the sociodemographic data on both participating partners. The data requested were: sex, age, present educational level, approximate income of the nuclear family, work and religious beliefs.

**Dating violence. Dating Violence Questionnaire-revised (DVQ-R)** [*Cuestionario de Violencia entre Novios CUVINO-R*] [50]. This instrument is composed of 20 items and aims to gather information on victimization and perpetration. They collect data on abusive behaviors or situations which can arise in a sentimental relationship, indicating their frequency on a Likert-type scale with five options: From 0 *Never* to 4 *Almost always*. The questionnaire (CUVINO-R) offers five differentiated forms of couple violence by homogenizing scores: *Detachment, Humiliation, Coercion, Physical Violence, and Sexual Violence.* The internal consistency for the total scale is α = 0.878 (Cronbach’s alpha). It is useful to mention that the instrument has numerous adaptations and validations not only in Spain, but also in many countries worldwide, showing an internal consistency for the five scales of about 0.64 and 0.74 (Cronbach’s alpha) and for the total scale α = 0.85 [50,51,52].

**Conflict resolution strategies.** The Conflict Resolution Styles Inventory (CRSI) [53] adapted for the Spanish population by Bonache et al., 2016 [42], comprises 13 items through which the studied subjects can evaluate with a range of 1 *Never* to 5 *Almost always*, indicating the frequency with which both they (CRSI-Self) and their partners (CSRI-Partner) use different conflict resolution strategies in determined situations. This version offers three conflict resolution factors: *positive resolution facing the conflict, negative involvement in the conflict, and lastly withdrawal from the conflict*. In this case, the internal consistency obtained for the total scale is α = 0.70 (CRSI-Self) and 0.74 (CRSI-Partner), both being adequate. Other authors have also found an internal consistency in this scale which is suitable for adolescent samples of 0.76 and 0.73 [54].

### 2.3. Procedure

First of all, this study was conducted in accordance with the Declaration of Helsinki (DH). Following the principles of DH, participants gave their voluntary informed consent to participate in the study. Second, participation of both partners of the couples was requested in a crowd-sourced way, and they were well-informed of participation anonymity and free voluntariness. Once both partners had confirmed their participation, an appointment was agreed with them, leading to the development of the study. At the start of the study, participants were explained the aims of the investigation, that is, they were informed that it was mainly to ascertain the dynamics of those couples that presented violent behaviors, as well as the conflict resolution strategies used when they were immersed in a situation of conflict, which did not mean that, specifically, their relationship presented these characteristics. Once both partners had confirmed their participation, they were invited to complete the above-described battery of instruments in which they had to answer about themselves and their partners. In all cases, the data were collected at a desk of the laboratory by a member of our research team who was trained to correctly apply these tools. Finally, once the battery of questions was completed, the data were properly saved in order to ensure anonymity of the data of participants.

The design of this investigation was via a survey with intentional non-probabilistic sampling, based on the requisites necessary for the attainment of the determined objectives of the research.

## 3. Statistical Analysis

The data were analyzed using IBM SPSS, version 25 (IBM, Armonk, NY, USA) and descriptive statistics are presented on the relevant study variables. Moreover, all the analyses were based on the “zero tolerance” criterion, that is, it was considered violent behavior when any of the values of the answer to the item were different from 0.

**Prevalence of violent behavior in adolescent and young couples.** First, with the objective of discovering which couples presented indices of violence, two study groups were formed. On the one hand, those couples who did not present any scores higher than 0 were assigned to the “*Non-violent couples*” group, and those who had indicated having perpetrated or suffered some of the violent behaviors described were assigned to the “*Violent couples*” group. Subsequently, transforming the five factors of violence (*detachment, humiliation, coercion, sexual, and physical*) into dichotomous variables (0 = no violence exists and 1 = violence exists) helped us to ascertain the prevalence of the factors of violence according to the two groups of couples.

**Prevalence of the occurrence of violence in violent couples.** After the study of the prevalence of violence in the couples, all the following analyses were carried out on the group of violent couples and the couples who had neither exerted nor suffered violence on the part of their partner were ruled out. Subsequently, with the dichotomous variables of each factor and each partner in the couple, using cross tabulation, the women who reported having perpetrated violence were analyzed labelling them as abusers, and the men were labelled as victims. This was repeated the other way round, that is, the men were labelled as abusers and the women as victims of violence. Once these analyses had been carried out, a descriptive chi-squared test (x2) was used to discover the possible significant differences that existed between being an abuser or victim according to sex. This analysis made it possible to establish four sample groups: women abusers, men abusers, women victims, and men victims. 

**Agreement on the occurrence and frequency of violence in violent couples.** We proceeded to examine the level of agreement between the two members, that is, the level or concordance between the information that was provided by the person who attacked and the information that was reported by the victim. This same analysis also led us to discover the level of disagreement between the two partners in the same couple. Moreover, the chi-square (x2) test was used and Cohen’s Kappa to interpret the consistency observed following the interpretation indices of (<0.40 = low level of agreement; 0.40–0.75 = acceptable agreement; >0.75 = excellent agreement). Lastly, the Wilcoxon non-parametric ranked test for related samples was used to see the existing differences and Cohen’s d effects sizes were calculated. 

**Prevalence of the use of conflict resolution strategies in violent couples.** In order to discover the use of conflict resolution strategies by violent couples facing situations of conflict, we transformed the three dimensions (*negative involvement in the conflict, positive resolution in the conflict, and withdrawal from the conflict*) into dichotomous variables *(0 = not used and 1 = used),* which helped us to ascertain the prevalence in the use of these strategies. Furthermore, an analysis for independent measures (Student’s *t* test) and Cohen’s effect size were calculated to understand the differences between the women and the men. Lastly, and as in the study of prevalence in the occurrence and agreement on the frequency of factors of violence, the same procedure was subsequently followed but with the conflict resolution strategies in the men and women from the sample who exerted violence towards their partners.

## 4. Results

### 4.1. Prevalence of Violent Behaviors in Adolescent and Young Couples

First, in order to attain the objectives determined in this study, we analyzed the obtained results using the zero-tolerance principle, that is, any answer different from 0 was considered as a violent act against the other partner. On this basis, the results indicate that of the 141 couples studied, only 29 (20.6%) did not mention any violent behavior in their dating relationships (*neither partner used violence*), compared with 112 (79.4%) who did identify themselves as violent couples (*one or both partners make use or have made use of violence*) (Table 2). Subsequently the prevalence was observed of the dynamics of the five factors of violence studied in this research (*Detachment, Humiliation, Coercion, Sexual and Physical*), and we found that in the couples considered violent, psychological violence was the most prevalent; first violence by detachment (87.5%) characterized by stopping talking to the partner, disappearing or ignoring the other’s feelings, followed by violence through humiliation (75.9%) that is, undervaluing, criticizing, ridiculing, or laughing at the other; and lastly, coercion (74.1%) holding the partner back or invading the other’s space. Apart from these factors of psychological violence which present the highest indices, no less important are the factors of sexual violence which were present in 47.3% of the couples in the sample and lastly physical violence in 30.4%. 

### 4.2. Occurrence of Violence in the Couples Depending on Sex (Man vs. Woman) and Role (Perpetration vs. Victimization) and Type of Violence (Detachment, Humiliation, Coercion, Sexual, and Physical)

Second, in order to analyze in depth how these dynamics were manifested in the group identified as violent couples (*n* = 112), we proceeded to examine the occurrence of violence in the couples depending on the sex, the role, and the type of violence. Analyses were done individually. What we found were high levels of psychological aggression and victimization both in the men and the women mostly obtaining significant differences. In particular, regarding perpetration, the factor of detachment is used by 68.8% of the men and 53.6% of the women, while violence through coercion of the other partner in 56.3% of the women and 50.9% of the men. In the case of violence through humiliation, men use it in a higher extent (53.6%) in comparison to women (51.8%). Regarding victimization, psychological violence also achieves the highest levels. In particular, men are who most suffer violence through coercion (57.1%) in comparison to women (47.3%) and women are who most suffer violence though detachment (58.9%) in comparison to men (50.9%). Consequently, male participants in this sample are victims of behaviors related to putting their love to test and even invading their personal space, with a higher proportion of women executing this type of behaviors. In contrary, women in this sample are victims of lack of support or affection by their male partners, these male partners being who perpetrate the highest level of this type of behavior. Regarding violence through humiliation, interestingly, men suffer it in a higher extent (56.3%) women (47.3%), which may be because, probably, men report suffering humiliation in a higher extent than women admit exercising. Finally, regarding occurrence of physical and sexual violence depending on sex and role, analyses yielded lower levels. Moreover, our data indicate that women use physical violence in a higher extent (19.6%) than men (12.5%) throw pushing, throwing objects or hurting their male partners, and the men suffer it in a higher extent (17%) than women do (10.7%). In contrast, men attack their female partners through sexual violence in a higher extent (20.5%) than woman (15.2%), throw touching, displaying sexual behaviors, and forcing their female partners to have sex with them and to undress without wanting. Likewise, women suffer sexual violence in a higher extent (24.1%) than men do (21.4%). However, the described sex-differences on sexual violence were not statistically significant in any role (Table 3).

### 4.3. Agreement on the Occurrence and Frequency of Violent Behaviors in the Couple Relationship 

Table 4 shows the results obtained in relation to the agreement that exists on the occurrence of violent acts in the couple relationship. First, it can be seen the level of agreement that exists between the violence that the man recognizes exerting on the female partner and the woman victim recognizes suffering, with statistically significant results and a low level of agreement (*Kappa* < 0.40). It can also be seen that the greatest agreement is obtained when it is a question of violence through coercion and physical violence. 

The same analysis was subsequently performed on the level of agreement between the violence that the women declared exerting on their men partners and the violence perceived by their men partners. Specifically, an acceptable level of agreement can be observed for the factors of psychological violence (*Detachment, Humiliation and Coercion*), however, the agreement decreases to a low level of agreement (*Kappa* < 0.40) when it is the case of sexual and physical violence.

Regarding the agreement on the frequency of dating violence perpetrated by women and men obtained from the Wilcoxon range test (Table 5), we can state that, when the man is the abuser there is more agreement on physical (80.09%) and sexual (69.98%) violence, followed by psychological violence which oscillates between approximately 34% and 44%, although these results were not significant. However, agreement when the woman is the perpetrator of the violence was only significant when it was a case of violence through humiliation (*Z =* 2.118; *p* < 0.05) and through coercion (*Z =* −2.840; *p* < 0.01), and the percentages of greater agreement were found in sexual violence (74.22%) and physical (71.9%) violence, although without showing significant differences. All the effect sizes were small or medium, except for the factors of coercion (*d =* 0.935) which showed high effect sizes. 

### 4.4. Prevalence of the Management of Conflict Resolution Strategies in Violent Adolescent and Young Couples

Table 6 shows the results on the prevalence of the use of conflict resolution strategies by the couples in the sample that reported violent behaviors and the possible differences found in relation to strategies employed in a situation of conflict with regard to sex. Here we can see the high use of positive conflict resolution strategies both in women (100%) and in men (97.3%) although there were no significant differences between sexes. With regard to the strategy of withdrawal, characterized by ignoring the person, staying silent, and behaving distantly, it was not found to be significant, with the men (73.2%) using this type of response to a greater extent. In the case of negative involvement when facing a conflict like throwing things, attacks, insults, and even loss of control among the adolescent and young couples, significant differences were found with a small effect size (*d* = 0.477) and, moreover, these data indicate that its use is more frequent in women *(t* (111) = 2.730; *p* < 0.01).

### 4.5. Agreement on the Frequency of Conflict Resolution Strategies in the Couple Relationship 

With regard to the agreement on the frequency of the use of conflict resolution strategies, the results show statistically significant discrepancies on the positive problem-solving strategy and the negative involvement in the conflict. The highest agreement between the two partners on the three dimensions of conflict resolution strategies (Table 7) was 14.86% when it was a question of the strategy of negative involvement in the conflict (*Z* = −3.540; *p* < 0.001) with a high effect size (*d* = 2.091). 

Lastly, with the aim of gaining in-depth knowledge about how to relate the violence found in adolescent and young couples and the conflict resolution strategies in men and women partner abusers (Table 8), we observed that those that perpetrated some of the five factors of violence, indicated that they used positive strategies with their partners. These data should be underlined as both men and women presented high percentages mainly in psychological type aggression; however, both reported using adaptive and positive strategies in conflicts with their partner. 

## 5. Discussion

Our society approaches couple violence from multiple perspectives, although the majority have always done so from one single vision, that of one partner in the couple. In the case of adolescents and young people, the literature tells us that in the past decade new forms of relating among the young people are given rise to new forms of violence [9,55]. In spite of having new modalities for executing violent behaviors they are still characterized by physical violence, sexual violence, and most frequently, psychological violence [56,57]. Given that the majority of studies only analyze one partner’s perception within the couple relationship which is a limitation in the research, it was considered to tackle this emerging state of affairs by doing the same analysis, but with both partner’s perception within the relationship, to thus compare the two visions and be able to work on all the associated risk factors [4]. The objective determined in this study was therefore to discover the level of agreement both on the occurrence and the frequency of violence between partners of adolescent couples and young adult’s partnerships, and, additionally, to ascertain the level of agreement between the partners of those couples on the frequency of the use of conflict resolution strategies by both partners when facing problematic situations. 

First, according to the principal of zero tolerance used, this investigation found that of the total number of couples who participated, 112 (79.4%) were identified as violent. Furthermore, our data show that the violence that they mainly use against their partner is psychological violence, with behaviors involving humiliation and the ridiculing of the other, insults, holding them back, invading their space and even failing to offer them affection as a sign of their anger. All of which coincides with the majority of the studies which reveal that psychological violence is the most predominant in the relations of adolescent and young adult’s couples, and in relation to the results of studies on the partner-actor which show mainly mutual psychological aggression [4,8,11,20,21,23]. 

To understand the dynamics of these dating relations in greater depth, prevalence of violence was studied according to the roles—perpetration vs. victim—within the couple and, specifically it was found that regarding psychological violence, the adolescent and young men were more prone to use violence against their women partners through detachment, followed by humiliation and coercion. Regarding the adolescent and young adult women, they use more violence against their men partners through coercion, followed by detachment and humiliation. However, the results of the metanalysis by Spencer, Toews, Anders, and Emanuels (2019) [58] show that coercion is considered a high-risk factor among men, that is, they use violence with the aim of exerting control over their women partners more frequently than women. With regard to more serious behaviors like sexual and physical violence, women use physical attacks, while men use sexual behaviors, although it should be underlined that these are not so prevalent, but they are present. These data coincide with studies by other authors who report in their investigations that women use physical violence to a greater extent and men are more prone to using sexual violence [4,14,18,31,59].

In this sense, it can be observed that the exercise of violence is common among boys and girls. Our data show that both boys and girls display an aggressor role and a victim role, and that, depending on the type of exerted violence, both members of the couple perpetrate or suffer abusive situations. Thus, in young people violence is an additional form of communication within the couple on the basis of which its members appear as aggressors and victims. These results are consistent with previous findings that indicate that there are no significant sex-related differences [20,23,26,28], which lead us to fulfill our first objective.

Within couple relationships, a key aspect to study is the level of concordance between the partners on the occurrence of aggressive behaviors. For this reason, we decided to investigate that extent of agreement when the women recognized exerting violence, and consequently the man reported being the victim of this violence, and vice-versa. In this respect, and according to our second objective, we found low levels of agreement, violence though coercion, and physical violence being the most acceptable levels of agreement. The lowest levels of agreement are to be found on sexual violence and violence through detachment. These results are partially in agreement with the results by Strandmoen et al. [28], which indicate a lower reliability regarding sexual violence, with couples showing a higher level of discrepancy. These results are consistent with the results by previous studies, which have found a decreasing trend in the agreement between the members of the couple when it comes to exerting sexual violence. This is due to the fact that the members of the couple do not recognize the violence that they exert on their partners and to the fact that it is more complex to accept that they are perpetrators or victims of that violence, with similar data being found with couples from the young population [31], and the adult population [28,29]. Regarding the lack of agreement on violence through detachment, it may be due to the fact that this type of psychological violence is not perceived as an act of serious gravity, as it does not cause visible damage and, therefore, it is thought that it does not entail as much impact on the other. On the other hand, violence due to humiliation also did not reach moderate levels of agreement, as did physical violence, which in previous investigations with adults also obtained low levels of agreement between the members of the couple [27,28]. Therefore, these results, in general, may be indicating that psychological violence, with the exception of coercion, is becoming normal in the interpersonal relationships of young couples. It could even be said that, in many cases, young people are not aware of the abusive situations in which they are immersed, which is known as non-perceived abuse.

The analyses of the agreement in the frequency of violent situations within the couple indicate that the highest levels of agreement occur in situations of sexual and physical violence, both for women with the role of aggressor and men with the role of aggressor. These results are in line with the previous research [28]. As it can be seen, in light of the results obtained both in the level of agreement on the occurrence and frequency of violent events, the information offered by the members of the couple confirms that there is a high level of disagreement. The fact that women report higher levels of violence than men may indicate that the level of agreement is affected by sex [31]. Regarding psychological violence, it is confirmed that it is the one most frequently reported by both members of the couple. However, despite the fact that significant differences have been obtained through many of our analyses, it can be said, from a global perspective, that younger couples do not show sharp differences in terms of the analyzed violence scales and the levels of agreement reached.

In view of the prevalence of violence that we encountered in the couple relations in our sample, we can conclude that there is widespread normalization of the use of violent dynamics especially of the psychological type of violence, given that independently of the abuser being a man or a woman, it is the one that shows the greatest percentages [12,20,49,60]. In our study, as in others, we can understand that this normalization of the aggressive patterns of the partners in the couple may be due to an inadequate ways of resolving situations of conflict, resorting to negative and violent involvement, which combined with the lack of experience and abilities in the interactions leads them to favor aggression and experience less subjective well-being [41,42,45,61]. Thus, we determined to ascertain the scope of the prevalence of conflict resolution strategies used by the violent couples according to the sex of the participants. Here we found contradictory data which may be biased by social desirability. These data make our third objective to be partially fulfilled. This is due to the fact that the partners indicated, without significant differences between them, that they more often use positive and adaptive strategies focusing on the problem, although it was also found that in couple conflicts the woman becomes negatively involved using personal attacks and experiencing loss of control, and the men tend to more use the strategy of withdrawing from the conflict, that is, while the women partners enter conflict, the men partners avoid it, which is frequent in young men abusers [54,62]. These last two are those that lead to the beginning of aggression as they enter into dysfunctional and aggressive dynamics [41,42,54]. However, most of the girls and boys indicated that they resolve their disagreements positively, focusing on the problem, in a constructive manner with alternatives that are accepted by both, data which should lead to much lower indices of violence. Regarding the agreement on the frequency of use of problem-solving strategies, it should be noted that the greatest level of agreement between both members of the couple is found in the use of negative involvement in the conflict, which leads to abusive behaviors within the relationship and is associated with high levels of victimization in adolescent and young couples [41,54].

Lastly, the explanation that currently exists is that the lack of abilities to manage conflictive situations is a factor of vulnerability for recognizing the mechanics of an abusive relationship, and even for being able to cope with cases of this type [63,64]. Studies based on conflict resolution show that there are differences probably caused by age between adolescents and young adults, with the former showing a greater deficit in abilities, making it difficult to cope with conflicts due to inadequate emotion regulation or incapacity to perceive the consequences of their actions. Furthermore, it is the women more than the men who use both psychological and light physical aggressive strategies in their conflicts [65]. 

With regard to the main limitations found in this study the following can be mentioned. First, the size of the sample, this methodological aspect could be enlarged and thus generalize the results on the frequent violent dynamics in adolescent and young couples in our population to different populations. Second, and related to the first, it could be interesting to extend the sample to include non-heterosexual couples as to work in the field of prevention we cannot forget an equally important part of the population. Third, social desirability in the responses of the participants as well as use of self-report measures as the only measure may leads to contradictory results. As a last limitation, more extensive work should be done on the relation between violence by adolescent and young couples and their used conflict resolutions strategies. 

## 6. Conclusions

As a general conclusion, the present investigation has focused on the study of both violent dynamics and conflict resolution strategies in adolescent and young couples, as well as the level of agreement between the partners on when abusive situations occur. The carried analyses have allowed us to verify the high prevalence of psychological violence in the studied couples, thus in agreement with previous research [4,11,20,23,25], a low level of agreement between the violent behaviors that one partner recognizes perpetrating and that the other recognizes suffering, thus in agreement with previous research [28,31,34], and higher agreement on the strategy of negative involvement in the conflict, which unfortunately encourages violent behavior and translates into lower subjective well-being [29,41,42,44].

On the other hand, we have carried out our study by including a relatively methodological novelty, which consists of using the information reported by both members of the couple -actor-couple- in relation to their experiences of violence in their relationship. Only few studies have studied this double perception when it comes to studying adolescent and young adult couples. In addition, we have included for the first time the study of occurrence of use of maladaptive problem-solving strategies as well as the level of agreement between the partners of the couples regarding their perceptions on the frequency of use of those strategies. Studying the double perception and the issue regarding problem-solving strategies is essential, if we want to know the prevalence of violence in young couples in a more accurate way. At the same time, it will help different professionals who are working to develop more effective prevention and intervention programs. A deeper understanding of partner violence from the perspective of the double perception will allow us to clarify the unknowns to promote much-needed social transformations.

It is to be outlined that the present study has contributed to show that the DVQ-R [50] provides professionals with a useful, reliable, and valid tool, which allows a quick evaluation, which accounts for the perpetration and victimization of violence in different types of violence. In this way, researchers will be able to access reports of the perpetration of violence and victimization of the same informant. In summary, it is a brief, easy-to-apply instrument that provides relevant information on different scales of violence -detachment, humiliation, coercion, physical, and sexual- from the role of aggressor and the role of victim. 

Finally, taking the findings of present study into account, we recommend trying to generalize the results to different target population and contextual realities by making future research efforts. 

## Figures and Tables

**Table 1:** Sociodemographic characteristics of the sample.

Characteristics		Women (*n* = 141)	Men (*n* = 141)
		$\bar{x}$	$\bar{x}$
Age	17–30	22.34	22.32
		***n* (%)**	***n* (%)**
Education	Secondary school High school Vocational Training	77 (54.6%)	95 (67.3%)
	University	64 (45.4%)	45 (31.9%)
Family Income	+2.500 €	28 (19.9%)	28 (19.9%)
	2.500–900 €	91 (64.5%)	98 (68.1%)
	−900 €	21 (14.7%)	17 (12.1%)
Work	Yes	45 (31.9%)	67 (47.5%)
	Not	96 (68.1%)	74 (52.5%)
Religious Beliefs	Not at all religious	53 (37.6%)	63 (44.7%)
	Moderately religious	55 (39%)	53 (37.6%)
	Very religious	33 (23.5%)	24 (17.1%)

**Table 2:** Prevalence of the types of violence (DVQ-R) in violent couples.

Type of Violence	Violent Couples—*n* and Percentage—by Type of Violence
**Detachment**	98 (87.5%)
**Humiliation**	85 (75.9%)
**Coercion**	83 (74.1%)
**Sexual**	53 (47.3%)
**Physical**	34 (30.4%)

**Table 3:** Prevalence of the occurrence of the types of violence (DVQ-R) in violent couples according to the roles of the partners: man aggressor-woman aggressor or man victim-woman victim.

	Aggressor (Man) *n* (%)	Aggressor (Woman) *n* (%)	*x^2^*	Victim Woman *n* (%)	Victim Man *n* (%)	*x^2^*
Total	101(90.2%)	93(83%)		95(84.8%)	95(84.8%)	
Detachment	77(68.8%)	60(53.6%)	0.512	66(58.9%)	57(50.9%)	8.107 **
Humiliation	60(53.6%)	58(51.8%)	20.458 ***	53(47.3%)	63(56.3%)	7.519 **
Coercion	57(50.9%)	63(56.3%)	20.687 ***	53(47.3%)	64(57.1%)	8.704 **
Sexual	23(20.5%)	17(15.2%)	2.675	27(24.1%)	24(21.4%)	0.179
Physical	14(12.5%)	22(19.6%)	9.341 **	12(10.7%)	19(17%)	5.822 *

Note. *n* = number of subjects; % = percentage of subjects; * *p* < 0.05 ** *p* < 0.01 *** *p* < 0.001.

**Table 4:** Agreement on the occurrence of the factors of violence (DVQ-R) in violent couples differentiated by role (man aggressor-woman aggressor/man victim-woman victim).

	Aggressor (Man)		Victim Woman			Agreement		Disagreement		*kappa*
	*n*	%	*n*	%	*x^2^*	*n*	%	*n*	%	
Detachment	77	68.8	66	58.9	12.774 ***	77	68.7	35	31.2	0.330
Humiliation	60	53.6	53	47.3	16.202 ***	77	68.7	35	31.3	0.377
Coercion	57	50.9	53	47.3	17.426 ***	78	69.6	34	30.4	0.393
Sexual	23	20.5	27	24.1	16.622 ***	88	78.6	24	21.4	0.383
Physical	14	12.5	12	10.7	17.280 ***	98	87.5	14	12.5	0.391
	**Aggressor Woman**		**Victim Man**			**Agreement**		**Disagreement**		***kappa***
	***n***	**%**	***n***	**%**	***x^2^***	***n***	**%**	***n***	**%**	
Detachment	60	53.6	57	50.9	10.291 ***	73	65.2	39	34.9	0.303
Humiliation	58	56.3	63	56.3	15.641 ***	77	68.8	35	31.3	0.372
Coercion	63	44.7	64	57.1	17.926 ***	79	70.6	33	29.5	0.400
Sexual	17	15.2	24	21.4	4.642 *	85	75.9	27	24.1	0.199
Physical	22	19.6	19	17	15.776 ***	91	81.2	21	18.7	0.374

Note. *n* = number of subjects; % = percentage of subjects; *x*^2^: Chi-squared; * *p* < 0.05, *** *p* < 0.001.

**Table 5:** Agreement on the frequency of the factors of violence (DVQ-R) perpetrated in the violent couples. Proportion of men and women who report greater violence than their partner (>), the same (=) and less violence (<).

	M > W		M = W		M < W		*Z*	*d*
	*n*	%	*n*	%	*n*	%		
Detachment	40	31.96	48	34.64	24	33.40	−1.633	0.485
Humiliation	32	28.56	56	43.02	24	28.42	−1.017	0.274
Coercion	30	29.93	54	41.03	28	29.04	−0.347	0.095
Sexual	13	15.08	82	69.98	16	14.94	−0.475	0.105
Physical	10	10.80	93	80.09	9	9.11	−0.537	0.112
	**W > M**		**W = M**		**W < M**		***Z***	***d***
	***n***	**%**	***n***	**%**	***n***	**%**		
Detachment	25	28.82	53	40.31	34	30.87	−1.265	0.353
Humiliation	19	26.84	57	45.3	35	27.86	−2.118 *	0.585
Coercion	26	25.98	45	35.63	40	38.39	−2.840 **	0.935
Sexual	9	12.50	84	74.22	16	13.28	−1.450	0.32
Physical	13	15.31	85	71.9	14	12.79	−0.247	0.054

Note. Wilcoxon Range test. M = man; W = woman; *n* = number of subjects; % = percentages of subjects; d: Cohen’s d; * *p* < 0.05, ** *p* < 0.01.

**Table 6:** Prevalence of the use of conflict resolution strategies (CRSI) by the violent couples according to sex.

	Woman			Man			*t*	*df*	*d*
	*n*	*%*	$\bar{x}$	*n*	*%*	$\bar{x}$			
Positive	112	100	1	109	97.3	0.97	1.748	111	0.235
Negative involvement	75	67	0.67	58	51.8	0.52	2.730 **	111	0.477
Withdrawal from the conflict	78	69.6	0.67	82	73.2	0.73	−0.706	111	0.112

Note. $\bar{x}$: Mean; t: Student’s *t*-test; *df*: degrees of freedom; ** *p* < 0.01; *d*: Cohen’s d.

**Table 7:** Agreement on the frequency of conflict resolution strategies (CRSI) in violent couples. Proportion of the couples that report a greater, equal, or lesser use of strategies.

	M > W		M = W		M < W		*Z*	*d*
	*n*	%	*n*	%	*n*	%		
Positive Resolution	42	41.60	14	1.53	54	53.87	−2.130 *	1.385
Negative involvement in the conflict	31	36.03	24	14.86	57	49.11	−3.540 ***	2.091
Withdrawal from the conflict	50	42.90	18	6.61	42	50.79	−0.024	0.011

Note. Wilcoxon’s range test. M = Man; W = Woman; *n* = Number of subjects; % = Percentages of subjects; d: Cohen’s d; * *p* < 0.05, *** *p* < 0.001.

**Table 8:** Prevalence of the occurrence of conflict resolution strategies (CRSI) of men abusers and women abusers in the couples.

	Positive Strategy		Negative Involvement Strategy		Withdrawal Strategy	
	Men Abusers in the Couple					
	*n*	%	*n*	%	*n*	%
Detachment	76	69.7	46	79.3	62	75.6
Humiliation	59	54.1	40	69	50	61
Coercion	56	51.4	41	70.7	46	56.1
Sexual	22	20.2	12	20.7	15	18.3
Physical	14	12.8	8	13.8	12	14.6
	Women Abusers in the Couple					
	*n*	%	*n*	%	*n*	%
Detachment	60	53.6	45	60	49	62.8
Humiliation	58	51.8	44	58.7	48	61.5
Coercion	63	56.3	50	66.7	49	62.8
Sexual	17	15.2	11	14.7	12	15.4
Physical	22	19.6	19	25.3	17	21.8

*n* = number of subjects; % = percentages of couples.

## Data Availability

The data that are reported here are available under request by members of the scientific community.
